# *Lacticaseibacillus casei* decrease long-chain fatty acids and most substances in an experimental model of intestinal mucositis

**DOI:** 10.1590/acb386723

**Published:** 2023-12-01

**Authors:** Stphannie Jamyla de Araújo Barbosa, Amanda Silveira da Silva, Maisie Mitchele Barbosa Oliveira, Susana Barbosa Ribeiro, Caroline Addison Carvalho Xavier de Medeiros, Leandro de Santis Ferreira, Francisco Ayrton Senna Domingos Pinheiro, Francisco Canindé de Sousa, Agnes Andrade Martins, Raimundo Fernandes de Araújo, Vinícius Barreto Garcia, Aurigena Antunes de Araújo

**Affiliations:** 1 Universidade Federal do Rio Grande do Norte – Postgraduate Program in Pharmaceutical Science – Natal (RN), Brazil.; 2 Universidade Federal do Rio Grande do Norte – Department of Pharmacy – Natal (RN), Brazil.; 3 Universidade Federal do Rio Grande do Norte – Postgraduate Program in RENORBIO, Department of Biophysical and Pharmacology – Natal (RN), Brazil.; 4 Universidade Federal do Rio Grande do Norte – Postgraduate Program in Oral Sciences – Natal (RN), Brazil.; 5 Universidade Federal do Rio Grande do Norte – Postgraduate Program in Functional and Structural Biology, Department of Morphology – Natal (RN), Brazil.

**Keywords:** Mucositis, Probiotics, Fatty Acids, Inflammation, Chemotherapy

## Abstract

**Purpose:**

To evaluate the long-chain fatty acid and major compounds levels in the feces after prophylactic oral use of *Lacticaseibacillus casei* in an experimental model of intestinal mucositis.

**Methods:**

Fifteen Swiss mice were randomly divided into three groups (n=5/group): The negative or positive control groups (n = 5) received saline orally for 18 days and an the intraperitoneal (i.p.) of saline or 5 Fluorouracil (450 mg/kg) in 15^th^ day, respectability. *L. casei* group received oral concentration of *L. casei* (1x10^9^ CFU/mL) for 18 days, the i.p. injection of 5-fluorouracil (450 mg/kg) in 15^th^ days. Tissue samples from colon and each small intestine segment were collected for histopathological analysis. Stool samples were collected. Fecal composition of long-chain fatty acids and sterols were analysed by gas chromatography-mass spectrometry on the 15^th^ and the 18^th^ day.

**Results:**

The mucosa layer of all small intestine segments of animals from L. casei showed well preserved epithelium and glands, without necrosis signs, but Goblet cells number decreased. Several long-chain fatty acids and sterols have been identified before and after in the groups. *L. casei* administration after 5-FU treatment reduced concentrations of linoleic acid (18:2) (p < 0.001) and oleic acid (18:1) (p < 0.001) in feces.

**Conclusions:**

*L. casei* prevented the mucosal damage associated with 5-FU-induced intestinal mucositis reduced long-chain fatty acid levels in the feces.

## Introdution

Intestinal mucositis is characterized by the deterioration of intestinal mucosal integrity, and it is directly linked to the adverse effects of 5-fluorouracil (5-FU), a widely used chemotherapeutic agent for various forms of cancer^1^. 5-FU acts by inhibiting DNA synthesis, leading to cell death, reduction of crypts and villi through enterocyte apoptosis, thereby affecting the intestinal mucosa^2^.

The Food and Agriculture Organization of the United Nations and the World Health Organization define probiotics as “live microorganisms which when administered in adequate amounts confer a health benefit on the host”^3^. They primarily consist of bifidobacteria and lactic acid bacteria, regulated by prebiotics (non-digestible food components)^4^.

Probiotics are used constantly to improve the homeostasis of internal microbiota to maintain the human intestinal health, and its bacterial strains have the potential to modulate colonic inflammation^5^.

The inflammatory response triggered by chemotherapy directly impacts the loss of the primary function of the intestinal epithelium, which is absorption. Essential substances for organismal homeostasis, such as vitamins, minerals, and lipids, have their absorption compromised due to the inflammatory condition^6^.

Fatty acid is an aliphatic chain carboxylic acid (COOH) produced when fats are broken down. They are poorly soluble in water (the longer the carbonic chain, the lower the solubility), and can be used as energy by cells. Fatty acids are formed by chains of carbon atoms that link to hydrogen atoms with an acidic radical at one end. Fatty acids can be in saturated form (the carbons have single bonds) or unsaturated form (with one or more double bonds). In the human body, it is through food that we consume fatty acids; they will be used as a source of energy for the functioning of our body^7^.

Linoleic acid (LA), an omega-6 fatty acid, and α-linolenic acid (ALA), an omega-3 fatty acid, are considered essential fatty acids because they cannot be synthesized by humans. Both omega-6 and omega-3 fatty acids are important structural components of cell membranes, serve as precursors to bioactive lipid mediators, and provide a source of energy. Long-chain omega-3 polyunsaturated fatty acids (PUFA) in particular exert anti-inflammatory effects; it is recommended to increase their presence in the diet^8^. 

Oleic acid (omega-9) reduced blood cholesterol and bad cholesterol (LDL). In addition, it had inflammatory effects (because it is rich in antioxidants), protected the heart, prevented cancer and slowed cell aging, in addition to helping to reduce platelet aggregation^9^. Oleic acid could be reported as an anti-inflammatory fatty acid playing a role in the activation of different pathways of immune competent cells^10^.

In a previous study conducted by our group^11^, it was observed that *Lacticaseibacillus casei* was able to reduce levels of pro-inflammatory cytokines and interleukins responsible for inflammation, thereby demonstrating immunomodulatory properties and beneficial effects on maintaining the brush border. Therefore, the objective of this article was to investigate the influence of *L. casei* of long-chain fatty acids (LCFAs) levels in an experimental model of 5-FU-induced intestinal mucositis.

## Methods

### Chemicals

5-Fluorouracil (Fauldfluor) was purchased from Libbs Pharmaceuticals LTDA. (São Paulo, SP, Brazil). *Lacticaseibacillus casei* was purchased from Farmafórmula (Natal, RN, Brazil). 

### Animals

Female Swiss mice (*Mus musculus*), weighing 25–30 g (mean age = 8 weeks old), were housed in polypropylene boxes and kept in controlled conditions of temperature (24 ± 2°C), relative humidity of the air (50 ± 5%), 12-h light/dark cycle and access to food and water *ad libitum*. All experimental protocols were approved by the Universidade Federal do Rio Grande do Norte Ethics Committee on the Use of Animals (No. 017/2019) and performed in accordance with the ARRIVE ethical guidelines. All methods were performed in accordance with relevant guidelines and regulations.

### Induction of experimental intestinal mucositis

Fifteen Swiss mice were randomly divided into three groups. The saline/negative control group received normal saline orally for 18 days, the intraperitoneal (i.p.) of saline in the 15^th^ day (n=5). The 5FU/positive control group received normal saline orally for 18 days, the i.p. injection of 5FU (450 mg/kg) in the 15^th^ day (n = 5). Three group received oral *L. casei* concentration of 1x10^9^ CFU/mL orally for 18 days, the i.p. injection of 5FU (450 mg/kg) in the 15^th^ days (n = 5). Animals were subsequently anesthetized on the 18^th^ day. The small intestines of the Swiss mice were then resected, and colon intestine tissue was collected were collected and fixed in 10% neutral-buffered formalin, dehydrated, and embedded in paraffin for histopathological analysis. Stool samples (all groups) on the 18^th^ day were collected immediately after defecation on a clean surface. The fecal samples were frozen immediately after collection and stored at -20°C until LCFA extraction. 

### Fatty acids in stool by gas chromatography mass spectrometer

Stool samples (all groups) on the 18^th^ day were collected immediately after defecation on a clean surface. The fecal samples were frozen immediately after collection and stored at -20°C until LCFA extraction and other apolar substances extraction. Approximately 100 mg of feces were weighed, placed in 1 mL of methanol, and submitted to mechanical stirring for 10 min. After that, the mixture was filtered and dried by SpeedVac. To LCFA esterification, 1 mL of 0.04 g/mL sodium methoxide was added to samples, that were kept at 65°C for 10 min with occasional shaking. For fatty acid methyl ester (FAME) extraction, a liquid-liquid extraction (LLE) was done adding to each mixture 500-μL water (one time) and 500-uL *n*-hexane (three times). The *n*-hexane phase was reunited and placed to a vial for a gas chromatography mass spectrometer (GCMS) analysis. Each extraction was replicated three times. 

Chromatographic gas analysis was carried out using an Agilent 8860 GC system equipped with a mass spectrometer (MS) model 5977B and an automatic sampler. A HP-5ms capillary column (30 m × 0.25 μm × 0.25 μm) was used. One microliter of the sample was injected into equipment in splitless mode. The initial oven temperature was 75°C, maintained for 5 min, then increased to 290°C at a rate of 6°C/min, and held for 20 min. The helium gas flow rate was set at 1 mL/min, and temperatures of the transfer line, ion source, and injector were set at 280 °C, 230 °C, and 250 °C, respectively. The ionization energy was 70 eV and data acquisition was done in scan mode for m/z 50−500. The identification of each substance was performed comparing the mass spectra to NIST (version 17) library data and retention index (table 1). To retention index calculation, a hydrocarbon mix standard solution was analyzed using the same GC-MS parameters.

**Table 1:** Overview of all substances identified by gas chromatography before and after 5-fluorouracil injection. For each, an indication of the retention time (RT), the compound name, class, and Kovats index.

Retention time (min)	Compound name	Class	Kovats index
26.70	Palmitic acid	Long-chain fatty acids	1,925
29.40	Linoleic acid	Long-chain fatty acids	2,094
29.50	Oleic acid	Long-chain fatty acids	2,100
29.86	Methyl stearate	Long-chain fatty acids	2,128
42.54	Cholesterol	Sterol	3,132
43.25	Lathosterol	Sterol	3,183
44.08	γ-Ergostenol	Sterol	3,241
44,23	Ergostanol	Sterol	3,246
44.60	Chondrillasterol	Sterol	3,269
45.65	γ-Sitosterol	Sterol	3,327
45.79	Stigmastanol	Sterol	3,335
46.66	α-Amyrone	Triterpenoid	3,381
47.15	α-Amyrin	Triterpenoid	3,405

Source: Elaborated by the authors.

### Histopathological analysis

Sections (5 μm thick) were obtained for hematoxylin and eosin (HE) staining and subsequent evaluation using light microscope (Olympus BH-2) at 200× magnification, by an experienced observer, in a blinded manner. The severity of SI inflammation (leukocyte infiltration and tissue damage in the intestinal parenchyma) was assessed by a descriptive analysis of the morphology of the segments of the duodenum, jejunum, ileum and colon.

### Statistical analysis

Data were analyzed using descriptive (mean and standard deviation) and analytical statistics using parametric tests such as analysis of variance (ANOVA), followed by a Bonferroni post-test and non-parametric Kruskal–Wallis’ test at a 5% significance level (Graph Pad Prism 8.01 software).

## Results

### Histopathological analysis

Negative control group (NC) showed absence of lesion. Histopathological of Duodenum, Jejunum, Ileum and Colon showed well preserved mucosa, with long villi and well-preserved crypts. The submucosa is intact, with normal submucosal glands and no signs of inflammatory infiltration or necrosis. Several goblet cells were found, especially in the Colon.

**Figure 1:**
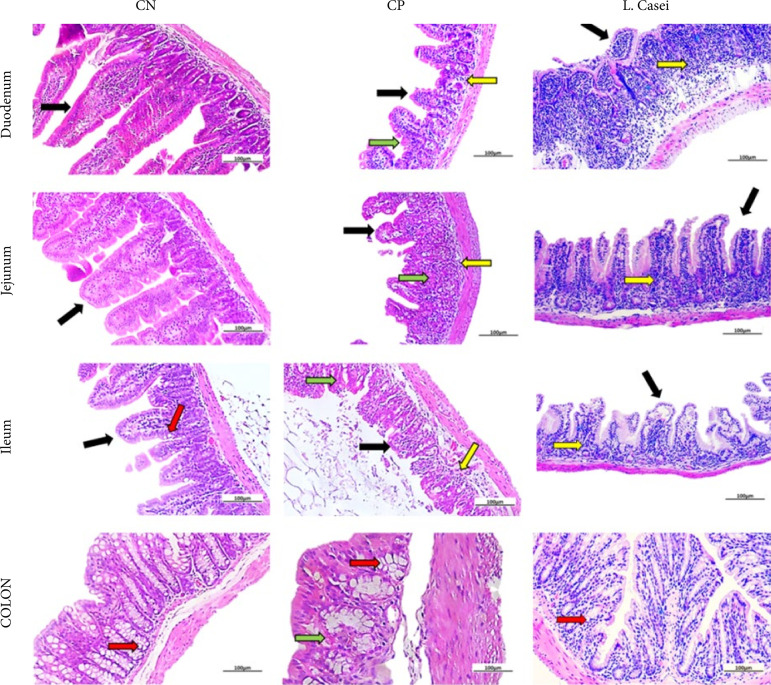
Description of histological characteristics of duodenum, jejunum, ilium and colon in CN, CP and *L. casei* group. CN: Histopathological of duodenum, jejunum, ileum and colon shows well preserved mucosa, with long villi (black arrows) and well preserved crypts. The submucosa is intact, with normal submucosal glands and no signs of inflammatory infiltration (yellow arrow) or necrosis (green arrow). Several goblet cells were found (red arrow), especially in the Colon. CP: 5-fluorouracil (5-FU)-injury group showed clear signs of damage, with short villi and small crypts and little evidence of goblet cells in duodenum’s mucosa. The submucosa is also injured, with signs of inflammatory infiltration and few submucosal glands. Both jejunum and ileum’s mucosa showed shortened villi and poorly preserved crypts in 5-FU-injured group, and just a few goblet cells were found in jejunum. Submucosal jejunum layer was heavily infiltrated by inflammatory cells (yellow arrow), with some signs of necrosis (green arrow). Short crypts were found in the colon’s mucosa, as well as necrosis foci. *L. casei*: no sign of inflammatory infiltrate was found in the submucosa layer of the colon. *L. casei* protected the colon and some small intestine segments against 5-FU-induced injury. Although inflammatory infiltration was still present throughout the whole small intestine submucosal layer (yellow arrow), the mucosa layer of all small intestine segments showed well preserved epithelium and glands, without necrosis signs. Goblet cells number decreased. No signs of injury were found in the colon segments.

CN: control negative control; CP: control positive control; L. casei: *Lacticaseibacillus casei*. Source: Elaborated by the authors.

PC: 5-FU-injury group showed clear signs of damage, with short villi and small crypts and little evidence of goblet cells in duodenum’s mucosa. The submucosa is also injured, with signs of inflammatory infiltration and few submucosal glands. Both jejunum and ileum’s mucosa showed shortened villi and poorly preserved crypts in 5-FU-injured group, and just a few goblet cells were found in jejunum. Submucosal jejunum layer was heavily infiltrated by inflammatory cells, with some signs of necrosis. Short crypts were found in the colon’s mucosa, as well as necrosis foci (Fig. 1).

*L. casei* protected the colon and all small intestine segments against 5-FU-induced injury as shown in Fig. 1. No sign of inflammatory infiltrate was found in the submucosa layer of the colon. *L. casei* protected the colon and some small intestine segments against 5-FU-induced injury as shown in Fig. 1. Although inflammatory infiltration was still present throughout the whole small intestine submucosal layer, the mucosa layer of all small intestine segments showed well preserved epithelium and glands, without necrosis signs. Goblet cells number decreased. No signs of injury were found in the colon segments.

### Analysis of apolar substances in a stool sample by GC-MS

**Figure 2:**
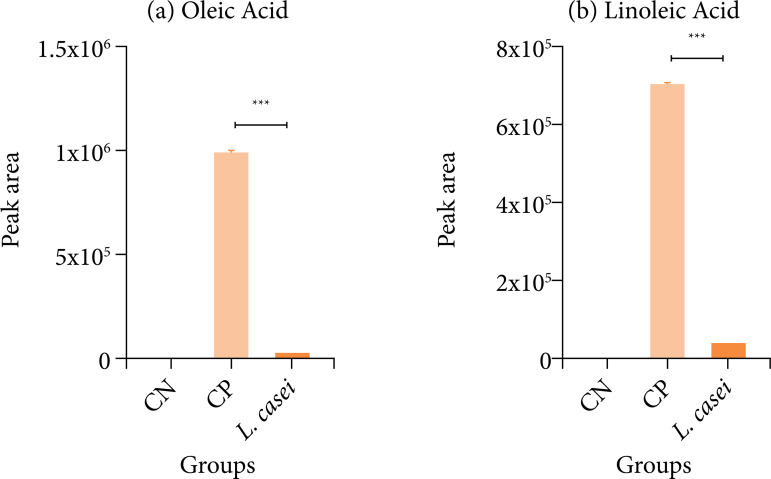
Gas chromatography mass spectrometry analysis identified the composition of long-chain fatty acids day 18 for the negative control (CN), positive control (CP) and *Lacticaseibacillus casei* groups. *Lacticaseibacillus casei* decrease oleic acid (p < 0.001) and linoleic acid (p < 0.001) after induction of intestinal mucositis by 5-fluorouracil.

Source: Elaborated by the authors.

Tables 2 and 3 show all substances identified by gas chromatography before and after for the negative control, positive control and L. casei groups. For each, an indication of the retention time (RT), the compound name, class, Kovats index, area. ± %relative standard deviation (RSD). GC-MS analysis identified the composition of apolar substances as long-chain fatty acids (LCFA), sterols and triterpens in day 18 for the negative control, positive control and L. casei groups, in view of the administration of 0.9% saline solution for the CN group and induction of intestinal mucositis by 5-FU for the CP and L. casei. L. casei was able to decrease oleic acid (p<0.001) and linoleic acid (p<0.001) after induction of intestinal mucositis by 5-FU, as illustrated in Fig. 2.

**Table 2:** Overview of all substances identified by gas chromatography after 5-fluorouracil injection. For each, an indication of the retention time, the compound name, class, Kovats index, and Area. ± %RSD.

Retention time (min)	Compound name	Class	Kovats index	CN (Area. ± %RSD)	CP (Area. ± %RSD)	*Lacticaseibacillus casei* (Area. ± %RSD)
26.70	Palmitic acid	LCFA	1.925	-	-	162.600 (± 0.9)
29.40	Linoleic acid	LCFA	2.094	-	251.480 (± 0.1)	100.931 (± 1.0)
29.50	Oleic acid	LCFA	2.100	-	314.497 (± 0.0)	70.483 (± 1.1)
29.86	Methyl stearate	LCFA	2.128	1.861.632 (± 1.7)	-	36.379 (± 2.0)
42.54	Cholesterol	Sterol	3.132	57.249.999 (± 0.3)	42.044.884 (± 0.1)	46.314.339 (± 3.0)
43.25	Lathosterol	Sterol	3.183	-	-	1.533.677 (± 0.5)
44.08	γ-Ergostenol	Sterol	3.241	5.349.676 (± 0.5)	4.702.568 (± 0.0)	10.676.351 (± 0.4)
44.23	Ergostanol	Sterol	3.246	-	-	5.250.666 (± 1.3)
44.60	Chondrillasterol	Sterol	3.269	-	-	3.720.188 (± 0.0)
45.65	γ-Sitosterol	Sterol	3.327	40.732.808 (± 0.0)	36.626.209 (± 0.1)	40.118.200 (± 3.0)
45.79	Stigmastanol	Sterol	3.335	-	-	8.222.519 (± 1.3)
46.66	α-Amyrone	Triterpenoid	3.381	-	-	1.717.520 (± 0.9)
47.15	α-Amyrin	Triterpenoid	3.405	-	-	1.884.442 (± 0.3)

LCFA: long-chain fatty acids; CN: negative control; CP: positive control; RSD: relative standard deviation (%). Source: Elaborated by the authors.

**Table 3:** Overview of all substances identified by gas chromatography before 5-fluorouracil injection. For each, an indication of the retention time, the compound name, class, Kovats index, Area. ± %RSD.

Retention time (min)	Compound name	Class	Kovats index	CN (Area. ± %RSD)	CP (Area. ± %RSD)	*Lacticaseibacillus casei* (Area. ± %RSD)
26.70	Palmitic acid	LCFA	1.925	-	-	-
29.40	Linoleic acid	LCFA	2.094	-	707.882 (± 0.4)	40.254 (± 0.5)
29.50	Oleic acid	LCFA	2.100	-	1.003.208 (± 0.5)	29.886 (± 0.4)
29.86	Methyl stearate	LCFA	2.128	-	416.361 (± 0.0)	-
42.54	Cholesterol	Sterol	3.132	30.182.411 (± 0.2)	57.209.074 (± 0.0)	43.071.492 (± 3.3)
43.25	Lathosterol	Sterol	3.183	-	-	1.085.832 (± 18.3)
44.08	γ-Ergostenol	Sterol	3.241	2.937.122 (± 0.0)	6.514.937 (± 0.0)	9.279.722 (± 2.6)
44.23	Ergostanol	Sterol	3.246	-	-	4.653.738 (± 2.1)
44.60	Chondrillasterol	Sterol	3.269	-	-	2.946.945 (± 4.5)
45.65	γ-Sitosterol	Sterol	3.327	22.646.952 (± 1.6)	52.750.942 (± 0.0)	31.973.951 (± 6.3)
45.79	Stigmastanol	Sterol	3.335	-	-	4.861.609 (± 21.5)
46.66	α-Amyrone	Triterpenoid	3.381	-	-	1.755.480 (± 2.9)
47.15	α-Amyrin	Triterpenoid	3.405	-	-	1.530.305 (± 1.1)

LCFA: Long-chain fatty acids; CN: negative control; CP: positive control; RSD: relative standard deviation (%). Source: Elaborated by the authors.

## Discussion

5-FU is an antineoplastic antimetabolite, and antimetabolites disguise themselves as purines or pyrimidines, getting incorporated into DNA during the S phase of the cell cycle, leading to disruption, impairment, and inhibition of the normal cell cycle. Gastrointestinal toxicity stands as one of the most frequent adverse reactions associated with cancer treatment, owing to the high occurrence of adverse clinical manifestations such as dysphagia, diarrhea, nausea, vomiting, and abdominal pain, due to the low selectivity of antineoplastic agents and the high rate of proliferation of the gastrointestinal tract epithelium^12^.

LCFAs are an important dietary component and contribute to various cellular functions and processes, including the synthesis of phospholipids, which play a crucial role in the structure, integrity, and function of cell membranes^13^. During the digestive process, LCFA molecules are dispersed in mixed micelles and, following digestion, are absorbed by enterocytes, especially in the jejunum and ileum segments, in which they are re-esterified and transformed into lipoproteins^14^.

LCFA is mainly transported to small intestinal epithelial cells through fatty acid transmembrane transporter proteins. For LCFA, studies have indicated that the absorption effect of long-chain saturated and unsaturated fatty acids in the small intestine is significantly different^15^. The absorption of palmitic acid and linoleic acid by small intestinal epithelial cells is similar, but the re-esterification rate of palmitic acid in small intestinal epithelial cells is much lower than that of linoleic acid, which is an important reason for the difference in bioavailability of these two fatty acids, and affects their absorption process^16^.

The findings of our previous study showed that the prophylactic use of *L. casei* in an experimental model of intestinal mucositis increased the number of CFU of *Lactobacillus* in the feces^11^. It has been observed that prior administration of *L. casei* to inflamed animals treated with 5-FU resulted in a significant reduction in tumor necrosis factor (TNF)-α, interleukin (IL)-6, and IL-1β levels. Immunohistochemical tests also revealed that pre-treatment with *L. casei* had a significant impact on the immunostaining of inducible nitric oxide synthase (iNOS) and TNF-α^11^. While small amounts of iNOS are necessary for homeostasis, large quantities, as produced upon iNOS activation, are detrimental^17^, highlighting the immunomodulatory potential of the probiotic. *Lacticaseibacillus casei* also played a role in preserving tight junctions, resulting in a significant reduction in the expression of nuclear factor kappa B (NFKB)-P65 and tool-like repectors (TLR)-4 genes. Furthermore, a positive effect was observed on the expression of MUC-2 and ZO-1 genes, which beneficially influence the components constituting the mucosal barrier, such as occludin and ZO-1^11^.

There are two main families of PUFAs that are relevant to human health, the omega-6 and the omega-3 PUFAs. In most diets, the PUFAs present in the highest amounts are LA and ALA. LA and ALA are not synthesized in animals and so are regarded as essential fatty acids^18^.

Dietary fat was recognized as a good source of energy and fat-soluble vitamins by the first part of the 20th century, but fatty acids were not considered to be essential nutrients because they could be synthesized from dietary carbohydrate^19^. This well-established view was challenged in 1929 by George and Mildred Burr, who reported that dietary fatty acid was required to prevent a deficiency disease that occurred in rats fed a fat-free diet. They concluded that fatty acids were essential nutrients and showed that LA prevented the disease and is an essential fatty acid. The Burrs surmised that other unsaturated fatty acids were essential and subsequently demonstrated that LA, the omega-3 fatty acid analog of LA, is also an essential fatty acid. During the 1970s LA was also recognized an essential nutrient for humans^19^.

Human beings evolved consuming a diet that contained about equal amounts of n-3 and n-6 essential fatty acids. Over the past 100-150 years, there has been an enormous increase in the consumption of n-6 fatty acids due to the increased intake of vegetable oils from corn, sunflower seeds, safflower seeds, cottonseed, and soybeans. Nowadays, in Western diets, the ratio of n-6 to n-3 fatty acids ranges from approximately 20–30:1 instead of the traditional range of 1–2:1. Studies indicate that a high intake of n-6 fatty acids shifts the physiologic state to one that is prothrombotic and proaggregatory, characterized by increases in blood viscosity, vasospasm, and vasoconstriction and decreases in bleeding time^20^.

Oleic acid could be a major element of the Mediterranean diet and presents totally different properties which will be helpful each within the immunomodulation, treatment, and bar of various forms of disorders like vas or response diseases, metabolic disturbances, skin injury and cancer, besides exerting outstanding role in drug absorption^21^.

In our study, *L. casei* was able to significantly reduced levels of oleic acid and LA. Evidence confirms that supplementation of LCFA has demonstrated the capacity to inhibit apoptosis of ileal mucosa cells, as compared to the group not supplemented with these fatty acids^17^. This once again underscores the impact of inflammation on lipid absorption, while the administration of *L. casei* appears to have alleviated this deficiency. An intact intestinal mucosa enables the efficient absorption of dietary components and enhances the activity of LCFA derived from it. This effect is distinctly observed in our study, as inflamed animals that were not pre-administered with *L. casei* exhibited substantial quantities of oleic acid and LA in their feces.

This study takes a step forward by investigating the effects of probiotic use on lipid absorption. *Lacticaseibacillus casei* administration after 5-FU treatment was able to reduce concentrations of LCFA in feces. The observed benefits may be attributed to the probiotics’ immunomodulatory effects and preservation of intestinal wall integrity, which directly impacts the absorption of various substances, including LCFA. 

## Conclusion

Oral administration of *L. casei* in the intestine against 5-FU-induced intestinal mucositis reduced LCFA levels in the feces.

## Data Availability

All data sets were generated or analyzed in the current study.

## References

[1] Elting LS, Cooksley C, Chambers M, Cantor SB, Manzullo E, Rubenstein EB (2003). The burdens of cancer therapy: Clinical and economic outcomes of chemotherapy-induced mucositis. Cancer.

[2] Bowen JM, Gibson RJ, Coller JK, Blijlevens N, Bossi P, Al-Dasooqi N, Bateman EH, Chiang K, Mooij C, Mayo B, Stringer AM, Tissing W, Wardill HR, van Sebille, Ranna V, Vaddi A, Keefe DMK, Lalla RV, Cheng KKF, Elad S (2019). Systematic review of agents for the management of cancer treatment-related gastrointestinal mucositis and clinical practice guidelines. Support Care Cancer.

[3] Sanders ME, Heimbach JT, Pot B, Tancredi DJ, LenoirWijnkoop I, Lähteenmäki-Uutela A, Gueimonde M, Bañares S. (2011). Health claims substantiation for probiotic and prebiotic products. Gut Microbes.

[4] Hill C, Guarner F, Reid G, Gibson GR, Merenstein DJ, Pot B, Morelli L, Canani RB, Flint HJ, Salminen S, Calder PC, Sanders ME (2014). Expert consensus document: The international scientific association for probiotics and prebiotics consensus statement on the scope and appropriate use of the term probiotic. Nat Rev Gastroenterol Hepatol.

[5] Pujo J, Petitfils C, Le Faouder P, Eeckhaut V, Payros G, Maurel S, Perez-Berezo T, Van Hul, Barreau F, Blanpied C, Chavanas S, Van Immerseel, Bertrand-Michel J, Oswald E, Dietrich G. (2021). Bacteria-derived long chain fatty acid exhibits anti-inflammatory properties in colitis. Gut.

[6] Sougiannis AT, VanderVeen BN, Davis JM, Fan D, Murphy EA. (2021). Understanding chemotherapy-induced intestinal mucositis and strategies to improve gut resilience. Am J Physiol Gastrointest Liver Physiol.

[7] Calder PC (2015). Functional Roles of Fatty Acids and Their Effects on Human Health. J Parenter Enteral Nutr.

[8] Saini RK, Keum YS (2018). Omega-3 and omega-6 polyunsaturated fatty acids: Dietary sources, metabolism, and significance: A review. Life Sci.

[9] Farag MA, Gad MZ (2022). Omega-9 fatty acids: potential roles in inflammation and cancer management. J Genet Eng Biotechnol.

[10] Carrillo C, Cavia Mdel, Alonso-Torre S. (2012). Role of oleic acid in immune system; mechanism of action; a review. Nutr Hosp.

[11] Barbosa SJ de A, Oliveira MMB, Ribeiro SB, de Medeiros CACX, Lima ML de S, Guerra GCB, Araújo RF, Sousa FC, Martins AA, Paiva DFF, Andrade RVS, Rebouças GSM, Brito GAC, Leitão RFC, Araújo AA (2022). The beneficial effects of Lacticaseibacillus casei on the small intestine and colon of Swiss mice against the deleterious effects of 5-fluorouracil. Front Immunol.

[12] Gifoni M. (2012). Mucosite e alterações de permeabilidade intestinal em pacientes portadores de câncer colorretal metastático tratados com 5-fluorouracil (5-FU) e irinotecano (CPT-11).

[13] Armand M, Borel P, Dubois C, Senft M, Peyrot J, Salducci J, Lafont H, Lairon D. (1994). Characterization of emulsions and lipolysis of dietary lipids in the human stomach. Am J Physiol Gastrointest Liver Physiol.

[14] Quaresma M, Damasceno S, Monteiro C, Lima F, Mendes T, Lima M, Justino P, Barbosa A, Souza M, Souza E, Soares P (2020). Probiotic mixture containing Lactobacillus spp. and Bifidobacterium spp. attenuates 5-fluorouracil-induced intestinal mucositis in mice. Nutr Cancer.

[15] Mu HL, Porsgaard T. (2005). The metabolism of structured triacylglycerols. Prog Lipid Res.

[16] Ockner RK, Pittman JP, Yager JL (1971). Differences in the intestinal absorption of saturated and unsaturated long chain fatty acids. Gastroenterol.

[17] Cerqueira NF, Yoshida WB (2002). Óxido Nítrico: Revisão. Acta Cir Bras.

[18] Djuric I, Calder PC (2021). Beneficial Outcomes of Omega-6 and Omega-3 Polyunsaturated Fatty Acids on Human Health: An Update for 2021. Nutrients.

[19] Spector AA, Kim HY (2015). Discovery of essential fatty acids. J Lipid Res.

[20] Simopoulos AP (1999). Essential fatty acids in health and chronic disease. Am J Clin Nutr.

[21] Piccinin E, Cariello M, De Santis, Ducheix S, Sabbà C, Ntambi JM, Moschetta A. (2019). Role of Oleic Acid in the Gut-Liver Axis: From Diet to the Regulation of Its Synthesis via Stearoyl-CoA Desaturase 1 (SCD1). Nutrients.

